# Stakeholder perceptions of cervical screening accessibility and attendance in Ireland: a qualitative study

**DOI:** 10.1093/heapro/daae072

**Published:** 2024-06-29

**Authors:** Sophie Mulcahy Symmons, Amanda Drury, Aoife De Brún

**Affiliations:** UCD Centre for Interdisciplinary Research, Education, and Innovation in Health Systems (UCD IRIS), School of Nursing, Midwifery and Health Systems, University College Dublin, Health Sciences Centre, 4 Stillorgan Road, Belfield, Dublin 4, Ireland; School of Nursing, Midwifery and Health Systems, University College Dublin, Health Sciences Centre, 4 Stillorgan Road, Belfield, Dublin 4, Ireland; School of Nursing, Psychotherapy and Community Health, Dublin City University, Collins Ave Ext, Glasnevin, Dublin 9, Ireland; UCD Centre for Interdisciplinary Research, Education, and Innovation in Health Systems (UCD IRIS), School of Nursing, Midwifery and Health Systems, University College Dublin, Health Sciences Centre, 4 Stillorgan Road, Belfield, Dublin 4, Ireland; School of Nursing, Midwifery and Health Systems, University College Dublin, Health Sciences Centre, 4 Stillorgan Road, Belfield, Dublin 4, Ireland

**Keywords:** cancer, screening, qualitative methods, participation, engagement, social determinants of health, cervical cancer screening, Ireland

## Abstract

Organized cervical screening programmes are commonplace in high-income countries. To provide an equitable cervical screening service, it is important to understand who is and is not attending screening and why. Promotion of screening and service improvement is not possible without recognition and identification of the barriers and needs of communities that are less engaged with screening. This study explored stakeholder perceptions of cervical screening attendance and accessibility in Ireland. Semi-structured interviews were conducted with 12 healthcare professionals, policymakers and academics. Interviews were conducted online in 2022. Reflexive thematic analysis was used inductively to generate themes, supported by NVivo. Three themes were developed: (i) getting the right information out the right way, (ii) acceptability and accessibility of screening and (iii) trying to identify and reach the non-attenders. Participants felt public knowledge of cervical screening and human papilloma virus was low and communication strategies were not adequate. Individual, cultural, structural and service-level factors influenced the accessibility and acceptability of screening. Identifying and reaching non-attenders was considered challenging and community outreach could support those less likely to attend screening. Stakeholder perspectives were valuable in understanding the complexities of screening accessibility and attendance from individual to service-level factors. Cultural competency training, inclusive language and visual cues in waiting rooms would support engagement with some populations who may be hesitant to attend screening. Collaboration with community organizations has opportunities to promote screening and understand the needs of those less likely to attend screening.

**Contribution to Health Promotion**

- This study contributed to understanding accessibility and attendance when quantitative data were limited.
- Current communication strategies may not engage women appropriately to their needs.
- Accessibility and acceptability of screening depend on individual, cultural and service factors.
- Engagement with community organizations could support understanding of non-attendance.

## BACKGROUND

### Call to make cervical cancer a rare disease

Cervical cancer is a preventable disease and screening plays a key role in early identification of cancer, reducing morbidity and mortality (Raffle *et al*., 2019). The World Health Organization (WHO) has proposed a strategy to make cervical cancer a rare disease in the next century (WHO, 2020). There are three components to this call to action: 70% of women (we use the term woman/women in this article to include any person with a cervix) screened by a high-performance test by the age of 35, and again by the age of 45; 90% of girls vaccinated by 15 years old, and 90% of cervical pre-cancers treated and invasive cancers managed (WHO, 2020). A high-performance screening test includes human papillomavirus (HPV) primary screening, which involves first testing for HPV infection, as it is present in most cervical cancers, rather than testing for abnormal cells first (Rebolj *et al*., 2019). HPV primary screening is being implemented globally, including in Ireland. Countries are beginning to announce goals to eliminate cervical cancer (WHO, 2023). The UK and Ireland have predicted elimination by 2040 and 2035 in Australia (Department of Health and Aged Care, 2023; National Screening Service, 2023; WHO, 2023). Challenges lie in achieving elimination equitably through equal access to treatment, vaccination and screening across and within countries, particularly where national vaccination and screening programmes are not established.

### Inequity in cervical cancer incidence and screening participation

The incidence of cervical cancer is highest in African countries and lowest in high-income countries (Singh *et al*., 2023). Cervical cancer incidence varied by 10 times between the highest [Eastern Africa (40 per 100 000)] and lowest [Western Asia (4.1 per 100 000)] regions with an average incidence of 13.3 per 100 000. Countries with a lower socio-economic Human Development Index had a higher incidence of cervical cancer (Singh *et al*., 2023). European evidence shows low socio-economic position (population and individual level) is associated with higher cervical cancer incidence (Mihor *et al*., 2020). Recent Irish literature demonstrated that women in the 25–59 age group, living in areas of high deprivation and urban areas have a higher incidence of cervical cancer in Ireland (National Cancer Registry Ireland, 2016).

International evidence reports that migrant and refugee women, women with disabilities, those identifying as LGBT+, and women of lower socio-economic backgrounds (area deprivation, low education, low income) are less likely to participate in screening (Kelly *et al*., 2017; Marlow *et al*., 2017; Willems and Bracke, 2018; Brzoska *et al*., 2020; Kellen *et al*., 2020; Saunders *et al*., 2021). In Ireland, the 2015–20 five-year average uptake of cervical screening is relatively high at 78.7% but is not equally distributed across demographic groups (National Screening Service, 2022). Nationally, there is limited data to identify what population subgroups have lower screening attendance as the national screening register only captures age and region-level data. Women over 50 years old have low participation in screening (National Screening Service, 2022). A recent systematic review endeavoured to identify populations in Ireland with lower attendance rates and found that participation appears to be lower for those in areas of deprivation, low education attainment, low socio-economic position, those identifying as LGBT+, in line with international evidence (Mulcahy Symmons *et al*., 2023). However, evidence varied how participation was measured and they captured a limited number of demographic factors, as such, robust methodologies and standardized reporting are required to clearly understand what factors influence participation in Ireland (Mulcahy Symmons *et al*., 2023). Contrary to international evidence, the only study in Ireland that collected data in relation to ethnicity, race or nationality found people of Irish nationality were less likely to have had a cervical screening test in the last 12 months in comparison to non-Irish nationality according to a 2019 health survey, however, it does not break down attendance into distinct nationalities (Central Statistics Office, 2020; Mulcahy Symmons *et al*., 2023).

### Cervical screening context in Ireland

Cervical screening was introduced as a national organized programme (CervicalCheck) in 2008. In 2018, the screening service conducted a retrospective audit identifying 221 women with false-negative results, which led to them developing advanced cancer that was not disclosed to them promptly. A tribunal was established to manage claims taken against the service and public confidence in the service was diminished. An independent review, the Scally Review, was conducted setting out recommendations for service improvement many of which have been implemented (Scally, 2018, 2022). HPV primary screening was introduced in 2020 but was stalled due to the COVID-19 pandemic. HPV primary screening is available for free to women and people with a cervix between the ages of 25 and 65. A national screening registry of women eligible for screening who have a Personal Public Service number. Details on the national screening register are obtained from the Department of Social Protection and from women when they register or when they go for their smear test. Women are sent a letter invitation generated from the national registry when they are due for screening; they are tested every 3 years between ages 25 and 29 and every 5 years from ages 30 to 65 and can attend any general practitioner (GP) practice, sexual health or family planning clinic. No research has been conducted to gather stakeholder perspectives on the screening programme since the introduction of HPV screening and the CervicalCheck crisis.

### Theory to understand screening participation

There are many theories to help explain variations in health and service use. The social determinants of health and socio-ecological model of health consider the individual, community, environmental, societal and system factors that affect health inequalities (Bronfenbrenner, 1977; Dahlgren and Whitehead, 1993). The Behaviour Change Wheel provides a framework to understand health behaviours through several components, capability, opportunity and motivation (COM-B) that can be targeted to change behaviour and improve health outcomes (Michie *et al*., 2011). These are useful lenses to elucidate who attends or does not attend screening, the influences on attendance and how the screening programme can be improved to ensure it is accessible to all.

### Aim

Understanding the distribution of participation and awareness of cervical screening can support and enhance targeted supports for women to make informed decisions about engagement with cervical cancer screening. Stakeholder contributions are important to understand problems and develop complex services and interventions (O’Cathain *et al*., 2019). Where national data are limited, qualitative insights from experts working in the field were deemed valuable to expose the context and state of the programme in relation to variations in screening attendance. This study aimed to explore the perceptions of expert stakeholders on cervical cancer screening attendance and accessibility in Ireland.

## METHODS

We undertook a qualitative descriptive study via semi-structured interviews to explore key stakeholder views of cervical cancer screening accessibility and attendance in Ireland. Ethical approval of this low-risk study was approved by the UCD Research Ethics Board (LS-23-35-Mulcahy-DeBrun). The consolidated criteria for reporting qualitative research (COREQ) was followed in this report (Supplementary Material S1) (Tong *et al*., 2007).

### Sample

Expert stakeholders in this study were those involved in planning or delivering the cervical screening service [healthcare professionals (HCPs), policymakers and academics]. Participants were purposively invited for interview and maximum variation sampling was used to ensure a range of perspectives were included (Palinkas *et al*., 2015). Stakeholders were identified through existing networks, identification of experts, outreach on the cervical screening sample-taker newsletter and snowball sampling.

### Data collection

All interviews were conducted by the lead researcher, an experienced qualitative researcher. All participants received an information sheet via email and signed a consent form before participating in an interview. No prior relationships existed between the interviewer and participants. The lead researcher has an MSc and health services research background, with expertise in population health.

The semi-structured interview guide consisted of questions on practice changes with the new HPV screening programme, what population groups were more or less engaged with screening, the barriers and enablers of attending screening as well as what supports may improve engagement and the service overall (Supplementary Material S1). The interview guide was pilot tested with a HCP, no changes were made to the guide. Interviews were conducted between February and May 2022 via Zoom and recorded. Interviews lasted 40–60 min. Interviews were transcribed verbatim by a third party and checked for accuracy by the lead researcher. Participants were sent their transcripts to review and in two cases revisions were made to the transcripts based on the participants’ requests. Sufficient information power was achieved through selection of participants with high levels of experience on the topic and depth of dialogue (Malterud *et al*., 2016).

### Analysis

Reflexive thematic analysis using an inductive, non-theory driven, approach was chosen to analyse the interviews to find patterns of meaning related to screening attendance and accessibility in the dataset (Terry *et al*., 2017; Braun and Clarke, 2022). First, the lead researcher listened to, read, and manually coded all interview transcripts. The lead researcher made summaries for each interview, incorporating reflective notes taken during and after the interviews. The lead researcher undertook a second round of coding more systematically using NVivo (12), incorporating insights from interview summaries and subsequently identifying codes that related to the research question. Independent academic researchers with qualitative experience from different professional backgrounds [oncology nursing (A.D.) and psychology and health systems research (A.D.B.)] coded two interviews supporting the critique and development of the initial analysis. The lead researcher refined and condensed initial codes and added new codes in iterations of analysis. The lead researcher grouped codes by similarity and meaning, supported by reflective journaling. These semantic and latent groupings were combined using post-it notes and mind mapping for initial theme development. The lead researcher engaged with existing literature on the topic, considering the parallels with the COM-B model, social determinants of health and socio-ecological model, which aided development of relationships within and between codes and themes (Bronfenbrenner, 1977; Dahlgren and Whitehead, 1993; Michie *et al*., 2011). The lead researcher shared theme summaries with the team to refine them, identifying some code repetition and other minor changes. After themes were developed, the lead researcher compared the themes to two transcripts where minor refinements were made. Participants were sent the themes for their input and feedback, but there were no suggestions to alter themes.

## RESULTS

**Fig. 1:**
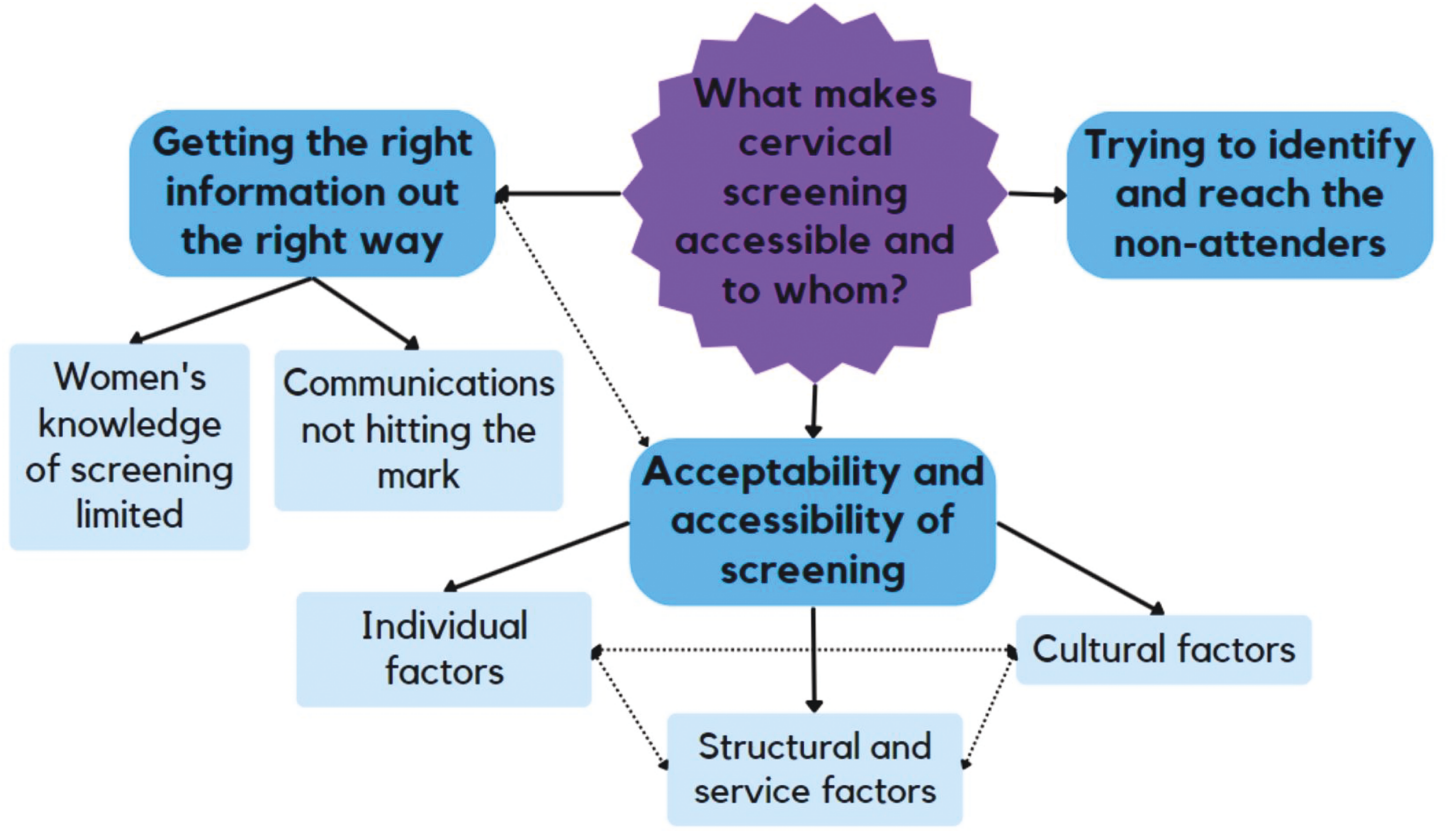
Map of themes.

Twelve interviews were conducted to explore those involved in planning or delivery of screening’ (policy, academic and HCPs) perspectives on cervical cancer screening attendance and accessibility in Ireland. All participants were experienced working in cervical screening delivery, implementation and research, and participant characteristics are described in Table 1. The three themes developed from the interview data are outlined in Figure 1: (i) getting the right information out the right way, (ii) acceptability and accessibility of screening and (iii) trying to identify and reach the non-attenders (Figure 1).

**Table 1:** Participant characteristics

Participant characteristics	Number (*N* = 12)
*Profession*	
Healthcare professional (HCP)	7
Nurse	4
GP	2
Colposcopist	1
Policymaker/academic	5
*Gender*	
Female	12
Male	0
*HCP place of work*	
Urban	6
Rural	1

### Getting the right information out the right way

Participants thought that some women had low awareness of cervical screening. Reasons for low awareness depended on discomfort discussing sexual health, low health literacy, low engagement with general health services and limited English fluency. Frequently mentioned was the belief that some women were unaware that they were eligible for the service, which included post-menopausal women, people in the LGBT+ community, single women who are not sexually active, in long-term relationships, nulliparous women, not engaged with gynaecological care or vaccinated against HPV.

*They [some people of lower socio-economic position] may not have the awareness of the importance of health prevention, prevention isn’t high on their radar. And then there are the older women who may never have come for a smear, or feel they no longer need to attend.* [P4—Policy/Academic]

Participants believed that women had limited knowledge of what screening meant (it is not diagnostic), what HPV was, and HPV’s link to cervical cancer, unless they were actively engaged in preventive healthcare. Since the introduction of HPV primary screening, HCPs felt they spent longer explaining screening, the link between HPV and cervical cancer and results before, during and after the exam.

*HPV is really complicated, so those conversations can be really complicated. And it’s, I think it’s taking me longer to explain the results … they knew what abnormal smear was, they don’t know what positive HPV is.* [P12—HCP]

Participants thought there was a misalignment between the national screening programme communication strategies and how women engage with health-related information. Participants had concerns that some women do not read the invitation letter and information leaflet fully as *‘It is too long. It’s off-putting’* [P3—HCP]. Participants believed it was essential that these materials were simple and easy to read as they were considered the first point of contact with the service. To improve communications, participants suggested refreshing media campaigns, using text or email invitations, using visuals to represent information and education at schools and universities. Younger women were believed to be more engaged with screening, partially due to their higher engagement with social media.

*She [a cervical screening advocate] has a Facebook page, and you know so people were engaging in that with her. [an online resource] that’s visual, that’s nice to look at, that you find out reliable information where the facts are you know, correct.* [P11—Policy/Academic]

HCP participants took opportunities to engage with patients about screening when they were overdue, emphasizing the need to be empathetic and understanding.

*I come across a young woman who has had a couple of babies, and they don’t know anything about cervical screening you know, I tell them about it and tell them it’s free and it’s very important that they get it done.* [P7—HCP]

Participants felt the 2018 CervicalCheck crisis was poorly managed by politicians and the health service and were disappointed by how it was portrayed in the media. Participants were protective of the integrity of the service, emphasizing the improvement made since the Scally review. Participants believed it eroded confidence in the service, with mixed views on whether or not it affected attendance.

*If you look at what happened in 2018 a lot of that was really ultimately you know the main failings were communication failures, so audit results and the meaning of an audit result was communicated*. [P2—Policy/Academic]

### Acceptability and accessibility of screening

Participants remarked that individual, cultural and structural factors increased or decreased accessibility and acceptability of screening attendance, rather than an active decision not to attend. The subthemes were informed by the socio-ecological model, COM-B and social determinants of health to explain the layers of influence on behaviour and attitudes to cervical screening.

There was a sense that the onus should be on women to make their health decisions. Stakeholders asserted that women must consent throughout the exam for screening to be acceptable. HCP participants emphasized the importance of developing rapport with the woman and ensuring they had autonomy throughout the exam. Participants believed the ability to check the register online gave women independence to book in when they were eligible. Participants considered that some women did not prioritize their health and well-being, putting their families first. Younger women were thought to be better at prioritizing themselves due to less competing priorities.

*I don’t think it’s out of any necessarily great awful aversion to having screening done, it’s just for a lot of women they’re extremely busy. And they’re not prioritising themselves.* [P6—HCP]

*The 25- to 30-year-olds seem particularly high [screening attendees], I think that’s certainly an impact from the comms we’ve done about screening and check the register and know where you’re at … so that she’s not waiting for a letter, that she’s not passive, that she’s kind of able to be an active participant in her health care.* [P8—Policy/Academic]

Participants remarked that women contended with various psychological factors that influenced screening acceptability and *‘almost every person comes in nervous’* [P12—HCP]. These factors included fear of potential illness, only thinking about attending health services if unwell, embarrassment about the intimate nature of the exam and fear of discomfort. Past negative experiences (either first-hand or second-hand stories from peers) were thought to affect attendance.

*It’s a very I suppose private thing … people just don’t want to do it and, would be embarrassed about going to have a test done and uncomfortable with it and have maybe had a painful experience in the past and don’t want to repeat that again*. [P11—Policy/Academic]

Women with positive experiences of screening were considered to attend more readily. Participants thought that women’s family and social networks promoted awareness of screening, allaying fears about the exam and subsequently normalizing screening.

*I suppose peers talking about the fact that they’ve had their smear, people coming and telling their friends they’ve had a smear and it was fine.* [P4—Policy/Academic]

Transport, childcare, literacy, inflexible appointment times, clinical rooms that were not universally accessible and equipment in general practices were perceived to reduce accessibility. Participants linked difficulty attending appointments to socio-economic circumstances and systemic inequalities.

*The harder-to-access women do tend to be women who would have more maybe social problems … You try to persuade them to come back in just to see us and they can find that difficult again for a number of reasons; childcare, transportation, cost*. [P1—HCP]

*There’s a bunch of patients that can’t make it in, physically into the clinic. And then my table doesn’t move. And so, I’ve, you know there’s been, even older people where we’ve had to get kind of creative with positioning and stuff because they can’t manage*. [P12—HCP]

Participants believed more choice with appointment times, female sample takers and being able to attend any clinic or GP made it easier for women to attend. HCP participants encountered challenges in completing screening in 15 min, given the complexity of information patients required. Participants believed that not rushing was essential to providing a positive care experience and their competencies and rapport with patients improved the acceptability of screening.

*Honestly don’t think you can do it in 15 minutes, you can, you know if you’re doing a procedure, you can do it, but if you’re giving them a proper experience you can’t do it*. [P6—HCP]

Participants were aware that unconscious biases and assumptions about their patients’ backgrounds and health literacy could prevent patients from feeling welcome and attending the practice. Participants suggested practices could be made more welcoming and inclusive by providing cultural competency training, visual cues in support of the LGBT+ community and informational leaflets in different languages.

*It was something like forty per cent of people in the LGBTQ community felt unwelcome in some practices which that is awful in this day and age. Actually, after I heard that you know what I did, I went and I got a rainbow lanyard.* [P7—HCP]

Culture was a recurring factor that participants felt added to the complexities of screening participation. Challenges included language, managing expectations arising from comparisons with screening systems in other countries, historical mistrust of health systems, religious backgrounds, different concepts of health, the belief that one should only access health services if unwell and loss of confidence in the service, including due to the 2018 CervicalCheck crisis (as described in theme ‘Getting the right information out the right way’).

*We know where people live, the culture they are raised in, the language they speak, their method of communication, you know, their understanding of screening, how they view the whole cancer area, and if they feel it’s worth engaging in this service because cancer can be prevented or cancer can be cured, so their attitudes and their beliefs come into play as well*. [P5—Policy/Academic]

### Trying to identify and reach the non-attenders

Despite their expertise and depth of experience, participants were unsure who the non-attenders of cervical screening were. They recognized the gaps in their knowledge of screening participation due to the lack of robust evidence and were challenged to think laterally about who attends screening. The use of the term ‘non-attender’ among some policy/academic participants was a de-sensitized view of those less likely to attend screening, however, participants were keen to understand who they are.

*So who are they [the non-attenders]? Where are they? Are they still active in the State, what’s the story with them? We don’t know a whole pile about that 20% and where they are and what they do. So, we’d like to know a little bit more about them. But the data is limited, and we have to do the best that we can within that.* [P5—Policy/Academic]

Ireland’s healthcare IT system was perceived as outdated and a major barrier to improving evidence on screening participation. Policy/academic participants hoped for improvements with the inclusion of indexing by address and the integration of unique health identifiers in screening registries. Participants felt better evidence had potential to target specific areas with low uptake rates with information campaigns and bespoke interventions. Participants relied on the limited research conducted in Ireland, international evidence, inferences from other screening programmes, application of the nine grounds of equality [under the Equality Act legislation (the Equality Acts protect individuals from discrimination based on their age, civil status, disability, family status, gender, housing assistance payments, membership of the Traveller community, race (colour, nationality or ethnic or national origins), religion, and sexual orientation)], and stakeholder engagement to determine whether participation was low in certain groups and communities. Participants felt they had a responsibility to reach out to vulnerable communities and engage with them.

*As we have become such a multi-cultural society in Ireland, I think we have a responsibility to outreach to the immigrant populations.* [P9—HCP]

Participants welcomed shifting priorities in health services and research where the patient and public voice are included. Collaborations between health services and community groups were perceived as contributing to successful interventions, which supported new collaborative projects with different communities and uptake of screening programmes.

*I was really impressed with [a charity for an ethnic minority population (Irish Travellers)] and we were chatting about how health care environments can be really intimidating for Traveller women so I think if you, you know, if you had outreach clinics and I think, there’s something about the kind of sisterhood and I think if women were together, so if you had a kind of a, maybe a chat first about why screening is important and then everyone could have their screening test and they also had a bit of a meet-up and a bit of a chat, that makes it a lot more interesting. … That takes care of the childcare issue.* [P8—Policy/Academic]

## DISCUSSION

### In the context of wider literature

Many studies investigate the factors influencing participation in cervical screening from the service user perspective, but few have explored other stakeholder perspectives. This study’s findings are informative in identifying challenges addressing inequality in cervical screening participation from a health system perspective. This study sheds light on strategies to promote screening and work with subgroups who are more likely to encounter challenges engaging with the service.

Themes one and two reflect current literature on the barriers and enablers of attending cervical screening from the woman’s perspective, which depend on awareness, psychological factors, accessibility and social context that continue to persist despite being known (Chorley *et al*., 2017; O’Donovan *et al*., 2021; Greenley *et al*., 2023; Wearn and Shepherd, 2024). Participants perceived women to either be actively engaged with screening or not be proactively engaged with screening due to time, lifestyle and other socio-cultural factors. Engaging with theory related to behaviour, attitudes and health in the analysis supports the alignment of results from this study with other research (O’Donovan *et al*., 2021; Wearn and Shepherd, 2024). Use of theory in turn informs development of interventions to promote screening that addresses specific barriers to attendance (Michie *et al*., 2011; O’Cathain *et al*., 2019). Subsequent research to this study will use behaviour theory and the social determinants of health to inform intervention development to promote cervical screening in Ireland.

Participants believed younger women were more likely to take part in screening as they were better able to prioritize their health and access health information proactively. This reflects the national screening data, where young women have high participation rates compared to women over 50 (CervicalCheck, 2022). From participants’ experience of individuals who have difficulty participating in screening, they identified people with caregiving responsibilities, those with chaotic lifestyles and struggles in daily living, those with low levels of English literacy, and those identifying as LGBT+: these groups reflect vulnerable social inclusion groups that are recognized to have additional specific needs (Health Service Executive, 2023). Those less likely to attend screening proved difficult to identify and define, despite acknowledgement of the need to engage them. Audits of cervical screening attendance in primary care practices may be a pathway to identifying demographic characteristics of those less likely to attend screening where unique health identifiers are yet to be integrated across health information systems. Expansion of demographics, such as socio-economic position and ethnicity, collected in the screening registry would enable an equitable approach to service improvement and health promotion.

### Opportunities to promote screening in the community

Women continue to have limited understanding of what cervical screening and HPV are (Chorley *et al*., 2017; O’Donovan *et al*., 2021). Participants felt GPs and nurses were key candidates to opportunistically provide information on screening. Within Ireland, there is a shift towards moving care into the community (Houses of the Oireachtas Committee on the Future of Healthcare, 2017). Identifying local HCPs and community workers who are known and trusted may have opportunity to promote screening during consultations. Community health programmes, such as smoking cessation, stress management and wellbeing, could integrate information about cancer screening. Identifying avenues to integrate education on screening within health consultations or other well-being initiatives could contribute to increased awareness and literacy on screening and potential participation in screening.

One key finding was the perceived mismatch between the way participants believed women wanted information versus the way it was delivered by the programme. Current practices such as letter-based invitations, paper leaflets and social media ads were thought to be less engaging for women. An interesting aspect of the data was the importance of women’s social networks in promoting or demoting screening (Chorley *et al*., 2017). Participants reflected that often women attended screening because their peers encouraged them to go, or they had a close family member affected by cancer. Contrarily, women could be disincentivized by peers’ negative past experiences of screening. A potential avenue could be to identify where women meet, e.g. online forums, coffee mornings, mother and baby groups, women’s sheds and target information sessions to these groups.

Community engagement is crucial to enhance understanding of barriers and enablers of participation in screening, particularly for under-screened and disadvantaged communities. Where evidence of screening participation and non-participation is limited (Mulcahy Symmons *et al*., 2023), engaging with community organizations that work with subgroups thought to have lower engagement with screening could enable specific and sensitive interventions that promote screening to be developed. Co-designed interventions enable people in a community to engage meaningfully with intervention development and produce outputs that are relevant and meaningful to target groups. In one study, a co-designed intervention to promote colorectal, breast and cervical screening among Muslim women in Scotland was acceptable to the population who felt their knowledge and perceptions of cancer screening had improved and had positive views on the co-design approach to developing the intervention (Christie-de Jong *et al*., 2022). Similarly, in Australia, a co-designed intervention to promote cervical screening among a culturally and linguistically diverse population was feasible and acceptable to the population (Power *et al*., 2022). Future work should engage meaningfully with communities to design interventions and materials that effectively promote screening.

This was the first study to assess perceptions of screening among those involved in the planning and delivery of screening since the CervicalCheck crisis. There are sustained concerns about public confidence in screening despite changes to the service. CervicalCheck have followed recommendations from the Scally report and collaborated with women affected by the crisis and the public to improve the programme and continue to work towards maintaining transparent, understandable communication pathways to repair public confidence (Scally, 2022). It will be important to continue to involve women in planning as recognizing and addressing the barriers for women at various system levels ensures a high-quality, equitable service.

### Strengths and limitations

This is the first study to get stakeholder perspectives on the screening programme since the introduction of HPV screening in Ireland and may be useful for countries planning to implement HPV primary screening programmes that are accessible to all communities. This study was conducted with stakeholders involved in the delivery of the service and did not interview women who are eligible for screening, however, findings aligned with international literature from women’s perspectives and bolstered the evidence in the field. We only interviewed one participant from a rural area, this limits our understanding from this perspective. No men were interviewed, limiting the diversity of perspectives, however, most sample-takers and stakeholders working in the delivery and planning of the service are female. We did not interview anyone who works in services dedicated to social inclusion groups who we recognize may have particular issues with screening; however, one participant did work in a deprived area and another worked part-time at a centre accommodating refugees.

### Practice implications

HCPs can strive to understand the woman’s complex and interlinking factors that influence attendance and recognize that these should be managed sensitively with a person-centred approach. Healthcare professionals may not be equipped to support these specific needs and require additional training through continued professional development and to provide the service in an understandable and sensitive way (Banerjee *et al*., 2020; O’Donnell *et al*., 2021; Debesay *et al*., 2022; Health Service Executive, 2023). Cultural and LGBT+ sensitivity and competency training can be provided to improve HCP skills. Healthcare professionals can opportunistically educate and conduct screening when appropriate. Community workers may be well placed to provide information about screening services in conjunction with other health and well-being information.

HPV self-sampling has been promising in promoting participation among women who are less engaged with screening (Di Gennaro *et al*., 2022). Consideration to implement self-sampling in Ireland must be in partnership with the public or communities who will be targeted to ensure acceptability and feasibility.

## CONCLUSION

The reasons for attending cervical screening are complex and dependent on a range of issues, from individual- to service-level factors. Stakeholder perspectives were valuable in providing a picture of screening accessibility and attendance and reported that opportunities to promote screening are ample in the community. Local HCPs and community groups provide a suitable setting to share information about screening, particularly where women come together in a safe and familiar environment. Challenges persist in identifying and engaging with those less likely to attend screening, however, collaboration with community organizations is key to co-design interventions and outputs that meet communities’ needs.

## Supplementary Material

daae072_suppl_Supplementary_Material
