# Supplementary material for: Stakeholder perceptions of cervical screening accessibility and attendance in Ireland: a qualitative study
Source: Health Promot Int. 2024 Jun 29;39(3):daae072. doi: 10.1093/heapro/daae072 (PMC11214098; doi:10.1093/heapro/daae072)
Supplement: daae072_suppl_Supplementary_Material [file daae072_suppl_supplementary_material.docx]

**Supplementary material for Stakeholder perceptions of cervical screening accessibility and attendance in Ireland: A qualitative study**

1. **COREQ Checklist**

**
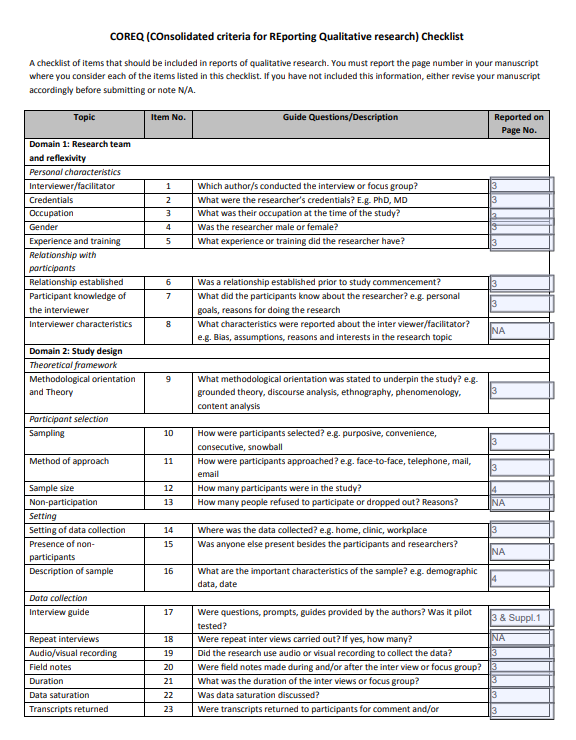
**


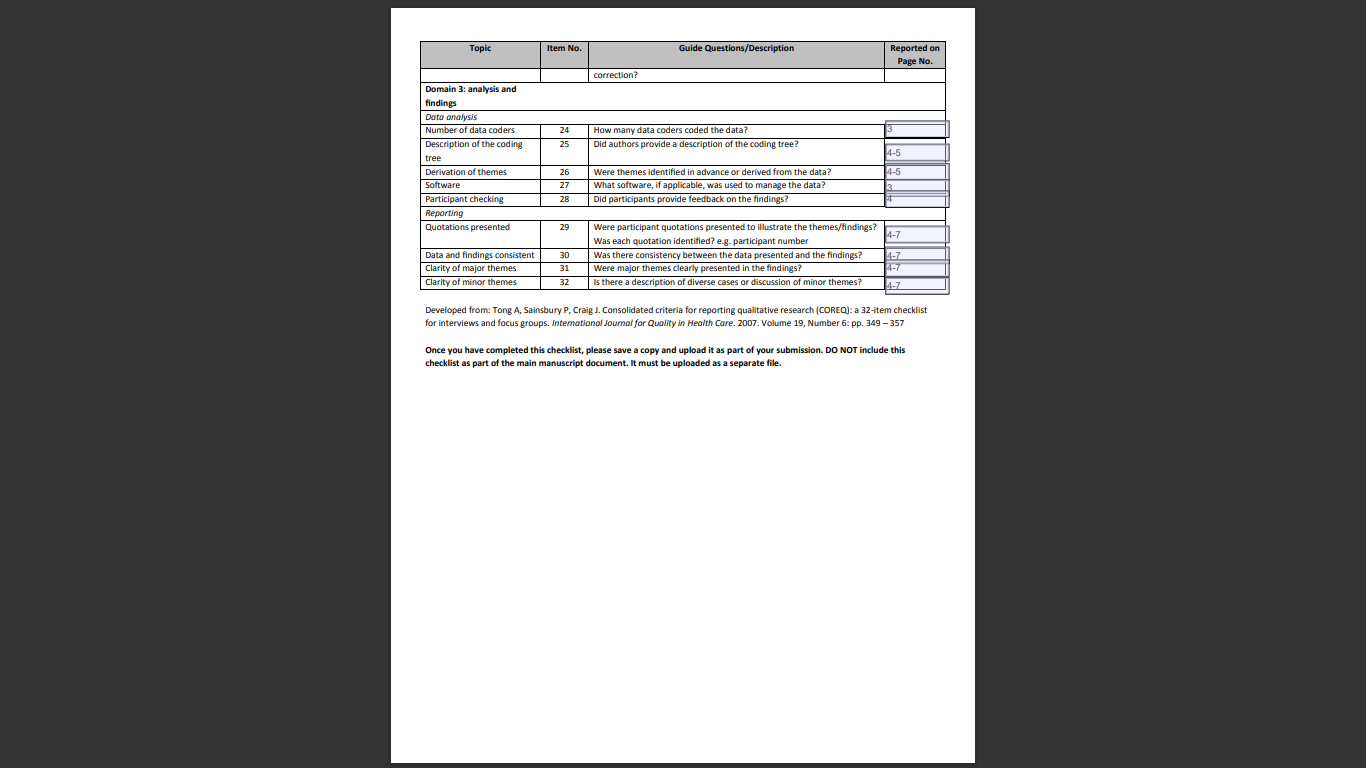


1. **Interview guide**

Greet the person, introduce yourself (PhD student, experienced interviewer) and explain the purpose of the interview is to better understand the screening process, the factors that influence (enablers/barriers) to attending cervical cancer screening and identify groups that are under-represented in screening. Explain that this is an early phase of a bigger PhD project that aims to collaboratively develop outputs that promote screening uptake.

Ask if there are any questions about the research. Check that you have consent. Remind them that this is voluntary, anonymous, contact details held separately to data collected, can withdraw any time and will be recorded. They are the expert, you might ask for clarification.

Start recording.

1. First, can you tell me a bit about yourself, your current role and its relation to cervical screening?
2. If Strategy/academia; How did you get involved in cervical screening research/monitoring? What has been your experience working in this role? Talk me through some of the research topics/projects?
3. If HCP; What has been your experience working in cervical screening/providing that service?/ Can you talk me through your last experience of taking a sample/screening/ a memorable experience? Do you have a sense of the patient experience?
4. The new HPV primary screening programme was rolled out in 2020. Since it was introduced, have you noticed any changes?
   1. Do you think anything about it is working particularly well or not so well?
5. Do you feel there is anything in general that influences engagement with the cervical screening programme?
   1. Has this changed since the introduction of the HPV screening programme?
   2. Have your colleagues noticed any differences/trends?
6. If HCP; What groups of people are served by your practice/organisation?/what’s your patient population?
7. From your perspective, are there particular groups of people who are more likely or less likely to engage with cervical screening?
   1. Why do you think that? Probe where they are getting info on/what are they basing answer on (media, research…)?
   2. Any other groups?
   3. If HCP; What are your interactions with this/these group/s like?
   4. If HCP, do those who attend screening differ from the general population you see in your practice? Differ from people with general access issues?
   5. Can you expand on that? – follow-up/probe, if academia, Ireland and international?
8. Do you think the message of screening is getting out to everyone? Who are communicated with but still don’t engage? Why? Who don’t get communicated with?
9. Are there any reasons why you think this/these group/s do or don’t engage?
   1. What do you think are the barriers to attending screening for this/these group/s?
   2. Individual or contextual or structural?
10. How do you know/find out who is and is not attending screening/where do you get your information?
11. If Strategy/academia; What do you think of how screening attendance data is measured? How do you think attendance could be captured/measurements and reporting improved? Opportunities to improve screening more broadly?
12. What do you believe might be needed to support those groups who are less likely to engage with cervical screening?
    1. If HCP; Has there been anything that’s worked or not worked for you in the past to support this/these group/s to attend screening?
    2. If HCP; Do you recommend screening to women who visit for other reasons? If yes, how would you approach it and what is their response? How do you discuss screening with women that don’t attend, have concerns about screening?
    3. If Strategy/academia; what projects on screening uptake have you undertaken/seen in the literature in the past? How did you decide on the project target pop?
    4. Have you seen any engagement strategies that have worked particularly well or not so well in the past? If yes, what did they involve/what happened?
13. If academia: where do you see the gaps in cervical screening research?
14. Is there anything you think we missed?
15. Any concluding remarks?
16. Do you have any questions for me?
17. Do you know anyone else who has a similar experience / performs a similar role / has similar expertise in the field? (email follow-up)

Thanks very much for speaking with me. Would you like me to hold onto contact details for the results of this research? Share transcript. Interested in continuing involvement? – explain advisory group.
